# Novel AI-Driven Precision Strategies in Diabetic Wound Healing: Immunomodulation and Advances in Smart Composite Nanocarriers

**DOI:** 10.3390/pharmaceutics18020252

**Published:** 2026-02-18

**Authors:** Yibin Zheng, Junshan Lan, Qian Huang, Qi Li, Yuting Liu, Bing Li, Xuan Wu, Qianxi Wang, Yongqi Liao, Xing Zhou, Zhipeng Teng, Jie Lou

**Affiliations:** 1 School of Pharmacy and Bioengineering, Chongqing University of Technology, Chongqing 400054, China; zhengyobi@stu.cqut.edu.cn (Y.Z.); lanjunshan@stu.cqut.edu.cn (J.L.); huangqian@stu.cqut.edu.cn (Q.H.); litchi77@stu.cqut.edu.cn (Q.L.); ytliu@stu.cqut.edu.cn (Y.L.); liibing@stu.cqut.edu.cn (B.L.); wuxuan@stu.cqut.edu.cn (X.W.); 18203087568@stu.cqut.edu.cn (Q.W.); liaoyqnet@stu.cqut.edu.cn (Y.L.); 2 Yunnan Key Laboratory of Stem Cell and Regenerative Medicine, School of Rehabilitation, Kunming Medical University, Kunming 650500, China; zhouxing@kmmu.edu.cn; 3 Department of Neurosurgery, Chongqing Hospital of Traditional Chinese Medicine, Chongqing 400011, China

**Keywords:** diabetic chronic wounds, immune microenvironment, smart composite nanocarriers, AI-driven precision therapy, real-time wound monitoring

## Abstract

Diabetic chronic wounds (CWs) represent a recalcitrant, difficult-to-heal pathological condition characterized by an imbalance of the immune microenvironment. Smart composite nanocarriers for immune regulation enable multi-targeted, spatiotemporally controllable synergistic interventions by responding to pathological signals such as reactive oxygen species (ROS), pH, and abnormal enzyme activity, thereby offering a novel pharmaceutical strategy to overcome the limitations of traditional single-target therapies. Artificial intelligence (AI) integrates clinical and biological data to predict healing risks, optimize treatment plans and nanocarrier design, and dynamically adjust strategies based on patient conditions, ensuring precision and personalized therapies. This paper systematically reviews the immunopathological basis of CWs, summarizes the design rationale and functional evolution of immune-modulating smart composite nanocarriers, and discusses an AI-enabled precision therapy framework from an interdisciplinary perspective. It aims to establish a theoretical foundation and research paradigm for constructing programmable drug delivery systems tailored to complex disease microenvironments, facilitating the transition of smart nanopharmacy from material-oriented to system-regulation-oriented approaches, and accelerating the clinically predictable translation of diabetic wound therapies.

## 1. Introduction

Diabetes mellitus (DM), as a major chronic metabolic disease worldwide, continues to escalate in prevalence and has emerged as a central challenge in global public health. CWs represent one of the most severe and debilitating complications of diabetes, primarily manifesting as foot ulcers [1]. The wound healing process is frequently arrested at the chronic inflammatory stage, leading to poor clinical outcomes and an increased risk of severe complications. This condition not only inflicts substantial physical and psychological trauma on affected patients but also imposes a significant socioeconomic burden, thereby constituting a critical bottleneck that urgently requires breakthroughs in current diabetes management strategies.

The core challenge in healing diabetic CWs lies in profound imbalances within the immune microenvironment. This manifests as a vicious cycle of “oxidative stress-immune dysregulation”, centered on dysregulated macrophage polarization. Persistent ROS accumulation, impaired macrophage phenotype switching, defective angiogenesis, and aberrant stromal remodeling collectively arrest wounds in the inflammatory phase, ultimately stalling the healing process at the chronic inflammatory stage [2,3]. Traditional treatments such as debridement, systemic antibiotic administration, negative pressure wound therapy, and topical growth factor application may alleviate local symptoms to some extent [4]. However, they suffer from inherent limitations including inefficient drug targeting, inadequate bioavailability, inability to reverse pathological microenvironment imbalances, and lack of personalized intervention adaptability. Consequently, clinical cure rates remain below 50% [5,6]. This falls far short of clinical treatment demands, urgently necessitating the development of precision therapeutic strategies based on novel mechanisms of action.

Smart composite nanocarriers, with their precisely designed physicochemical properties and modular functions, enable targeted drug accumulation at diabetic wound sites and on-demand release responsive to pathological microenvironments (e.g., ROS, pH) [7]. By integrating multiple actions including antioxidant, immunomodulatory, and pro-reparative effects, these carrier systems aim to directly intervene and remodel the imbalanced wound immune microenvironment, offering a novel precision pharmaceutical strategy for diabetic wound treatment [8]. Such nanocarriers are typically constructed from multi-component material platforms (e.g., hydrogels, nanozyme-based systems, and hybrid nanocomposites), whose therapeutic performance is governed by key material parameters including stimulus responsiveness, structural stability, and drug–matrix interactions [9]. However, traditional nanoparticle designs often rely on empirical experimental iterations, resulting in lengthy development cycles, high costs, and difficulty in addressing the individual heterogeneity of patient wounds—such as varying degrees of inflammation, infection status, and metabolic levels. This mismatch between material design and wound heterogeneity represents a major barrier to efficient clinical translation.

AI and big data technologies provide critical support for the clinical translation and precision treatment of nanocarriers [10]. By integrating multidimensional data from clinical, molecular biology, and pharmaceutical domains, AI algorithms enable intelligent prediction of wound healing risks, optimization of nanocarrier performance, and personalized treatment plan formulation [11,12]. In the context of highly dynamic wound pathophysiology and pronounced interpatient variability, machine learning has been increasingly applied to DFU monitoring and prognosis, while sensor-integrated smart dressings offer a practical foundation for AI-assisted closed-loop wound management [13,14]. This approach effectively addresses the challenges posed by the heterogeneity of diabetic wounds, driving a revolutionary shift in treatment paradigms from empirical approaches toward precision pharmacology.

Based on this, this paper systematically reviews the pathological characteristics of diabetic CWs and the molecular mechanisms underlying immune microenvironment dysregulation. It comprehensively summarizes the classification, pharmaceutical functional design, and core immune regulatory targets of immunomodulatory smart composite nanocarriers. It emphasizes the pivotal role of AI and big data in optimizing nanocarrier pharmaceutical properties and developing personalized treatment strategies. The paper thoroughly examines key challenges in advancing this field from fundamental pharmaceutical research to clinical translation, while outlining future interdisciplinary integration directions. This comprehensive review aims to provide systematic guidance for advancing precision pharmaceutical treatment technologies for diabetic wounds and facilitating their clinical translation.

This review was based on a structured search of the PubMed, Scopus, and Google Scholar databases using the keywords “diabetic chronic wounds,” “smart composite nanocarriers,” “immune modulation,” and “AI-driven therapeutic strategies,” covering publications published between 2019 and 2026. After screening and evaluation, 131 peer-reviewed publications were included, primarily focusing on immunomodulatory mechanisms, smart nanocarrier design, and emerging AI-assisted therapeutic strategies in diabetic wound healing. In addition, clinical trial registries, including ClinicalTrials.gov, were independently consulted to provide complementary insight into ongoing or recently completed clinical studies, thereby enhancing the understanding of the current translational landscape in this field.

## 2. Core Pathological Features of Chronic Wounds in Diabetes Mellitus

The intractable nature of chronic diabetic wounds stems from profound disruption of the integrated “metabolism–inflammation–microcirculation–neural regulation” network. Hyperglycemia and advanced glycation end products (AGEs) activate signaling cascades such as RAGE/NF-κB, thereby inducing persistent inflammation and oxidative stress that accelerate extracellular matrix (ECM) degradation [15,16,17]. Concurrently, ischemia–hypoxia and neuroregulatory imbalance synergistically exacerbate deterioration of the local wound microenvironment, leading to coordinated dysfunction of reparative cells and immune cells and ultimately obstructing critical healing pathways [18,19]. Elucidating the interactive mechanisms within this highly interconnected pathological network provides a fundamental theoretical basis for the development of targeted and mechanism-driven therapeutic strategies, as summarized in Table 1.

### 2.1. Metabolic Dysregulation and Accumulation of Advanced Glycation End Products

Hyperglycemia represents the initiating and central pathological driver of impaired healing in chronic diabetic wounds, with its detrimental effects largely mediated by the abnormal accumulation of AGEs [28]. AGEs disrupt the wound repair microenvironment through two principal mechanisms. First, AGEs bind to their receptor RAGE, thereby activating downstream signaling pathways such as NF-κB, which sustain chronic inflammation and oxidative stress and establish a self-amplifying pathological loop. Second, AGEs directly cross-link structural proteins, including collagen, within the ECM, resulting in matrix stiffening that impedes cell migration and angiogenesis [29,30]. Recent studies further demonstrate that AGEs can directly inhibit angiogenesis by disrupting the interaction between integrin α5β1 and galectin-3 (Gal-3), consequently suppressing focal adhesion kinase (FAK) signaling [31]. Moreover, topical administration of Gal-3-based hydrogels has been shown to effectively promote wound healing. Moreover, a positive feedback loop involving hyperglycemia, AGEs accumulation, and oxidative stress has been clearly established [32]. Based on these findings, targeting the AGEs–RAGE signaling axis and its downstream pathological networks—including inflammation, oxidative stress, and ECM remodeling—through precise local intervention emerges as a critical therapeutic paradigm. Localized delivery strategies, such as hydrogel-based systems, enable direct modulation of the wound microenvironment, thereby breaking the vicious cycle of diabetic wound chronicity and shifting therapeutic focus from systemic glycemic control toward localized precision repair.

### 2.2. Vascular Lesions and Microcirculatory Disorders

The pathogenic mechanisms underlying vascular lesions and microcirculatory disorders in diabetic wounds have been elucidated at multiple levels. Hyperglycemia, dyslipidemia, and AGEs collectively drive a maladaptive “metabolic–inflammatory” phenotype, exacerbating endothelial injury and lipid deposition through mitochondrial ROS bursts and persistent activation of the NLRP3 inflammasome [33,34,35]. At the microcirculatory level, the diabetic milieu directly impairs endothelial cell proliferation and migration by suppressing PFKFB3-mediated glycolysis, ultimately leading to insufficient neovascularization [36,37]. The resulting hypoxic microenvironment further aggravates repair failure by triggering aberrant metabolic programs in resident cells, characterized by excessive glycolysis and histone lactylation, which disrupt intercellular communication and reparative signaling [38]. Concurrently, hypoxia-associated cues drive macrophage polarization toward dysfunctional inflammatory states, promoting sustained release of pro-inflammatory mediators that inhibit tissue regeneration [39]. Collectively, these findings indicate that future therapeutic strategies should prioritize the development of multifunctional and integrative interventions capable of simultaneously disrupting the “metabolic–inflammatory” vicious cycle, correcting endothelial metabolic dysfunction (e.g., PFKFB3 signaling), and reprogramming immune cell phenotypes, particularly macrophages. Smart nanocarrier-based delivery systems and responsive biomaterials offer a promising avenue to fundamentally dismantle the pathological closed loop of “ischemia–hypoxia–inflammation–repair failure” that characterizes CWs.

### 2.3. Neuropathy and Sensory-Motor Dysfunction

One of the core mechanisms by which diabetic neuropathy impairs wound healing lies in the loss of neuropeptide-mediated signaling, including substance P and calcitonin gene-related peptide (CGRP), resulting from sensory nerve damage. These neuropeptides play essential roles in regulating local angiogenesis, immune cell activity, and epithelialization [40,41]. Restoration of neuropeptide signaling through targeted interventions has therefore emerged as a promising therapeutic strategy. For example, the α-lipoic acid–metformin elastomer patch developed by Song et al. significantly upregulated CGRP and nerve growth factor (NGF) expression, thereby simultaneously promoting angiogenesis and inducing macrophage polarization toward the reparative M2 phenotype, achieving coordinated nerve repair and immune microenvironment remodeling [42]. Similarly, strategies combining conductive hydrogels with electrical stimulation have been shown to directly promote nerve regeneration while guiding macrophage polarization toward the M2 phenotype [43,44]. Collectively, these advances underscore that future therapeutic paradigms must extend beyond structural nerve repair and instead actively regulate the “neural–immune–vascular” interactive network. The most promising direction involves the development of multifunctional biomaterials—such as smart patches and conductive hydrogels—that spatiotemporally integrate neurotrophic, immunomodulatory, and proangiogenic signals, thereby reconstructing the wound microenvironment in a coordinated manner and elevating therapeutic goals from localized symptom control to systemic regenerative modulation.

### 2.4. Organizational Repair Barriers and Cellular Dysfunction

**Figure 1:**
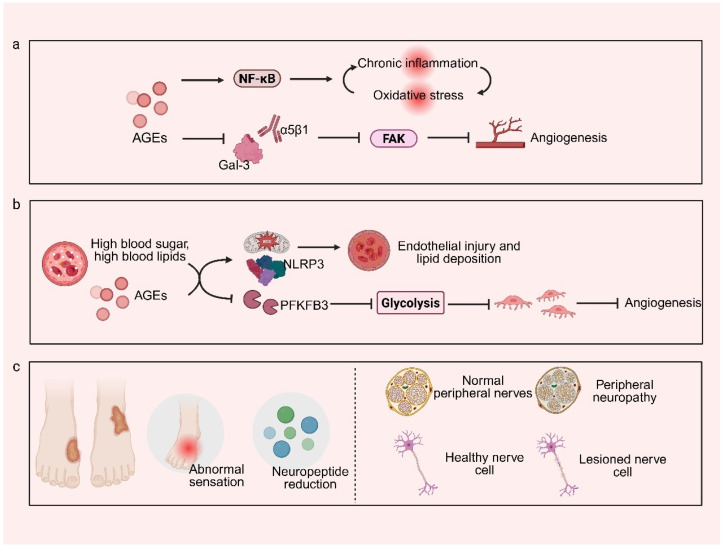
Schematic illustration of core pathological mechanisms in diabetic CWs. (**a**) AGEs accumulation activates NF-κB-mediated inflammation and oxidative stress and disrupts α5β1–FAK signaling, thereby suppressing angiogenesis. (**b**) Hyperglycemia and hyperlipidemia induce NLRP3 inflammasome activation and inhibit PFKFB3-dependent glycolysis, leading to endothelial dysfunction and impaired angiogenic capacity. (**c**) Diabetes-associated peripheral neuropathy, characterized by reduced neuropeptide release and structural nerve damage, disrupts neurovascular crosstalk and delays wound healing. Created in BioRender. Huang, Q. (2026). BioRender.com/qgoexux.

In diabetic CWs, the coordinated function of key reparative cell populations—including fibroblasts, keratinocytes, and vascular endothelial cells—is severely disrupted. Fibroblasts exhibit not only impaired proliferation, migration, and collagen synthesis but also aberrant activation of endoplasmic reticulum stress pathways, which drive excessive secretion of mediators such as monocyte chemoattractant protein-1 [45,46]. Through the “fibroblast–immune cell crosstalk” axis, this process induces abnormal activation of natural killer cells and suppresses macrophage polarization toward the M2 phenotype, effectively trapping the wound in a persistent inflammatory state [47]. Simultaneously, keratinocyte migration and re-epithelialization are markedly delayed, while endothelial cells display compromised lumen formation and impaired angiogenic capacity [48]. In response to this multilayered cellular dysfunction, targeted micellar systems based on click chemistry have demonstrated precise interventional potential. Representative examples include restoring fibroblast function via targeting the PAR-2 receptor or reshaping macrophage polarization through glycosylation-based modification strategies [49]. Taken together, these findings indicate that effective treatment of diabetic CWs must transition from single-cell or single-pathway correction toward systematic reprogramming of the entire reparative network. Smart nanocarriers engineered through functionalized designs such as click chemistry offer multi-target recognition and synergistic delivery capabilities, providing a critical technological pathway for simultaneously regulating the pathological interactions among fibroblasts, immune cells, and vascular endothelial cells. Such integrated strategies hold promise for dismantling self-perpetuating pathological cycles and reinitiating stalled healing processes, as illustrated in Figure 1.

## 3. Key Features of the Immune Microenvironment in Chronic Wounds Associated with Diabetes

The core mechanism underlying impaired healing in chronic diabetic wounds lies in profound dysregulation of the local immune microenvironment, a complex pathological state that has increasingly become a central focus of investigation. Accumulating evidence indicates that this dysregulated microenvironment does not arise from isolated immune abnormalities but rather constitutes a multidimensional and highly interconnected network of imbalance. At its core is the abnormal prolongation and failure of resolution of the inflammatory phase, accompanied by functional and phenotypic dysregulation of key immune cell populations, including macrophages, neutrophils, and lymphocytes. These immune alterations are tightly intertwined with persistent oxidative stress and aberrant ECM remodeling, forming a mutually reinforcing pathological loop. This vicious cycle—characterized by disrupted immune response timing, impaired intercellular communication, and maladaptive tissue microenvironmental cues—not only perpetuates tissue damage and inhibits reparative progression but also provides critical mechanistic entry points for the development of innovative therapeutic strategies aimed at restoring immune homeostasis in diabetic wounds.

### 3.1. Immune Dysregulation Characteristics in CWs Associated with Diabetes

#### 3.1.1. Prolonged Inflammatory Phase and Excessive Accumulation of Pro-Inflammatory Factors

**Figure 2:**
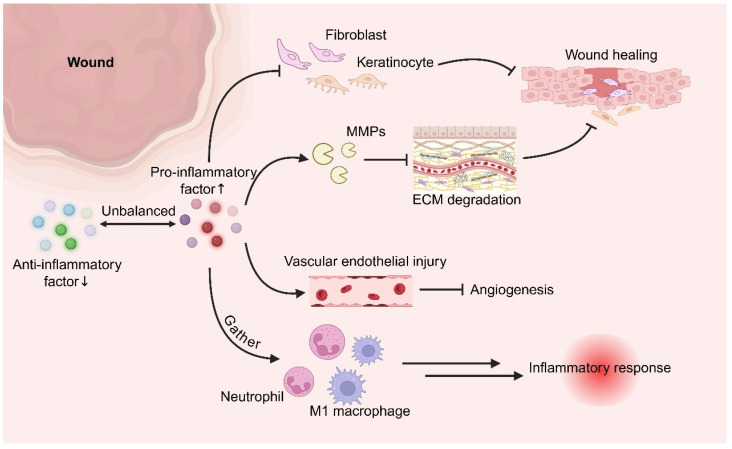
Inflammatory imbalance-driven immune dysregulation in diabetic CWs. Inflammatory imbalance sustains chronic inflammation, promotes neutrophil and M1 macrophage accumulation, induces MMPs-mediated ECM degradation, and impairs angiogenesis. Red symbols represent pro-inflammatory mediators, while green symbols indicate anti-inflammatory components. Upward arrows (↑) denote upregulation, and downward arrows (↓) indicate suppression. Created in BioRender. Huang, Q. (2026). BioRender.com/s08e4ol.

The most prominent immunopathological feature of chronic diabetic wounds is a persistent and unresolved inflammatory response, resulting in a sustained “chronic inflammatory state”. The expression levels of pro-inflammatory mediators—including TNF-α, IL-6, IL-1β, and IFN-γ—are significantly elevated in diabetic wound tissues compared with acute or non-diabetic wounds and remain abnormally high for prolonged periods. In contrast, the expression of anti-inflammatory cytokines, such as IL-10, IL-4, and TGF-β1, is relatively insufficient, leading to a pronounced imbalance between pro-inflammatory and anti-inflammatory signaling [50,51,52]. Extensive evidence suggests that this imbalance is closely associated with the failure of macrophages to effectively transition toward the M2 reparative phenotype. Pharmacological interventions that promote M2 polarization, such as resveratrol administration, have been shown to significantly reduce the expression of pro-inflammatory mediators including TNF-α and IL-1β, thereby partially restoring immune balance [53]. Excessive accumulation of pro-inflammatory factors not only suppresses fibroblast and keratinocyte proliferation and migration—thereby impairing granulation tissue formation and re-epithelialization—but also induces overexpression of matrix metalloproteinases (MMPs), leading to excessive degradation of the ECM and disruption of tissue architecture. Moreover, sustained inflammation exacerbates endothelial cell injury, inhibits angiogenesis, and promotes massive infiltration of neutrophils and M1 macrophages, further amplifying the inflammatory cascade [54,55]. Mukai et al. demonstrated that in db/db diabetic mouse models, TNF-α mRNA levels in wound tissues were significantly elevated from days 7 to 14 post-injury, while IL-6 expression showed marked increases on days 11 and 14 compared with wild-type controls [56]. These findings provide direct evidence that persistent overexpression of pro-inflammatory mediators is positively correlated with delayed wound closure in diabetic settings. Collectively, prolonged inflammation and excessive accumulation of pro-inflammatory factors drive progressive tissue damage, ultimately entrapping diabetic wounds in a self-sustaining non-healing state, as illustrated in Figure 2.

#### 3.1.2. Immune Cell Dysfunction and Phenotypic Imbalance

##### Imbalance in Macrophage M1/M2 Polarization

In diabetic wounds, macrophages exhibit a pronounced M1-dominant residency phenotype, characterized by sustained secretion of inflammatory mediators such as TNF-α, IL-1β, and MMP-9, which directly exacerbate inflammation and disrupt reparative processes [57,58]. This aberrant polarization state is closely linked to mitochondrial dysfunction, as hyperglycemia and chronic inflammation drive excessive accumulation of mitochondrial ROS. Elevated ROS levels activate the MyD88/NF-κB signaling pathway, thereby stabilizing the M1 phenotype and inhibiting the transition toward the M2 reparative state [59,60]. Targeting macrophage polarization has therefore emerged as a critical therapeutic strategy. For example, the SIRT3 agonist SZC-6 enhances the expression of the mitochondrial fusion protein Mfn2 and reduces ROS accumulation, thereby suppressing NF-κB activation. This intervention significantly decreases the proportion of M1 macrophages while promoting expression of M2-associated markers [61]. Similarly, bioactive dermal substitutes (BADS) loaded with bone marrow-derived mesenchymal stem cells represent a mechanistically defined strategy to correct macrophage M1/M2 polarization imbalance. These constructs have been shown to downregulate M1-associated TNF-α and MMP-9 while concomitantly upregulating anti-inflammatory mediators such as IL-10 and TGF-β3. At the mechanistic level, this immunomodulatory effect is achieved through targeted inhibition of the TNF-α/NF-κB signaling axis, as evidenced by reduced phosphorylation of IKKα/β, IκBα, and NF-κB p65, thereby attenuating pro-inflammatory macrophage activation and favoring a reparative phenotype [62]. Collectively, these interventions promote non-contractile epithelial healing by modulating the M1/M2 macrophage ratio, thereby enhancing granulation tissue formation, collagen deposition, and angiogenesis. Based on these mechanistic insights, future therapeutic strategies for diabetic wounds should prioritize the development of smart delivery systems capable of spatiotemporally precise regulation of macrophage polarization. An ideal approach should integrate the following features: (i) mitochondrial-targeted delivery technologies, such as triphenylphosphine-based nanocarriers, to restore macrophage metabolic plasticity; (ii) co-delivery of SIRT3 agonists and NF-κB inhibitors to synergistically suppress inflammatory signaling and promote reparative phenotype switching; and (iii) engineered cell–material interfaces, including biomimetic scaffolds with localized release of stem cell-derived exosomes, to continuously guide macrophage polarization toward the M2 phenotype. Such multimodal strategies aim not only to correct polarization imbalance but also to systematically reconstruct the wound repair microenvironment.

##### Neutrophil Clearance Impairment, Abnormal NETs Accumulation, and Lymphocyte Subpopulation Dysregulation

Dysregulation of both innate and adaptive immune compartments synergistically exacerbates immune imbalance in diabetic wounds. Neutrophils display particularly pronounced dysfunction, characterized by delayed apoptosis and excessive NETosis, leading to pathological accumulation of neutrophil extracellular traps (NETs) [63,64]. Clinical studies have reported significantly elevated levels of NET-associated markers, such as citrullinated histone H3, in diabetic foot ulcer tissues, which correlate positively with ulcer severity [65]. Mechanistically, hyperglycemia activates peptidylarginine deiminase 4 (PAD4), thereby driving excessive NETs formation. NETs subsequently activate TLR9 signaling in endothelial cells, triggering the PAK2–Merlin axis, suppressing the Hippo–YAP pathway, and inducing endothelial–mesenchymal transition, which collectively impair angiogenesis. In parallel, cell-free DNA released from NETs persistently amplifies inflammatory signaling through the TLR9/NF-κB pathway, further reinforcing chronic inflammation [66]. Intervention studies have confirmed that targeted NETs clearance, using agents such as DNase I or PAD4 inhibitors, represents a mechanism-driven strategy to correct impaired neutrophil clearance and pathological NETs accumulation, thereby suppressing endothelial–mesenchymal transition and promoting vascular regeneration and wound closure.

Adaptive immune dysregulation further compounds this pathological state. T lymphocytes in diabetic wounds exhibit a reduced CD4^+^/CD8^+^ ratio and heightened secretion of pro-inflammatory cytokines, including IL-2, IFN-γ, and TNF-α [67]. Concurrently, diminished activation of B lymphocytes results in insufficient production of IL-10, weakening humoral immune regulation and pathogen clearance while further destabilizing immune homeostasis [68]. These abnormalities in neutrophil and lymphocyte function are tightly interconnected, collectively sustaining a chronic inflammatory microenvironment that impedes effective tissue repair.

**Figure 3:**
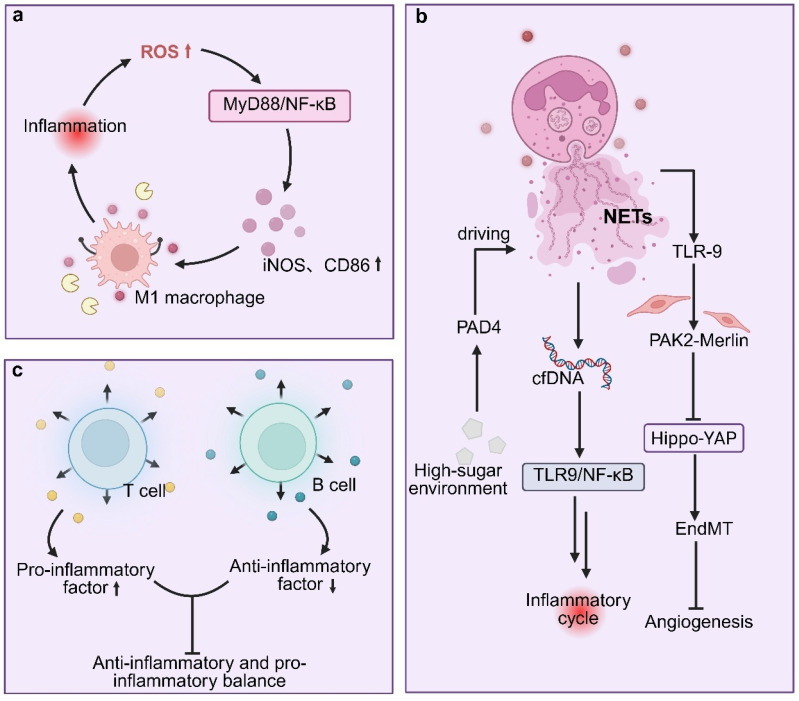
Immune cell dysfunction-mediated inflammatory amplification in diabetic CWs. (**a**) ROS–MyD88/NF-κB signaling promotes M1 macrophage polarization and sustains inflammation. (**b**) PAD4-dependent NETs formation activates cfDNA–TLR9 signaling and suppresses angiogenesis. (**c**) Dysregulated T- and B-cell responses disrupt the pro-/anti-inflammatory balance. Upward arrows (↑) indicate increased expression or activation, while downward arrows (↓) indicate suppression. Solid arrows represent activation or promotion, and blunt-ended lines indicate inhibitory effects. Red shading represents inflammatory activation. Created in BioRender. Huang, Q. (2026). BioRender.com/hizjhk6.

Based on these observations, therapeutic strategies targeting immune dysregulation in diabetic wounds must move beyond single-cell or single-pathway interventions toward integrated, multi-target immunomodulatory approaches. In particular, synergistic therapies capable of simultaneously regulating innate immunity (e.g., inhibition of NETosis) and adaptive immunity (e.g., restoration of T/B cell balance) are required. For example, smart delivery systems responsive to wound-specific microenvironmental cues could be engineered to co-deliver PAD4 inhibitors alongside IL-10 mimetics or regulatory T cell-inducing agents, thereby suppressing excessive inflammation while enhancing reparative immune responses in a spatiotemporally controlled manner. The core objective of such strategies is to disrupt pathological positive feedback loops among immune cell populations, offering a fundamentally new immunological paradigm for reversing chronic non-healing in diabetic wounds. The mechanisms underlying immune cell dysfunction and inflammatory amplification in diabetic wounds are illustrated in Figure 3.

### 3.2. The Vicious Cycle of Oxidative Stress and Immune Dysregulation

**Figure 4:**
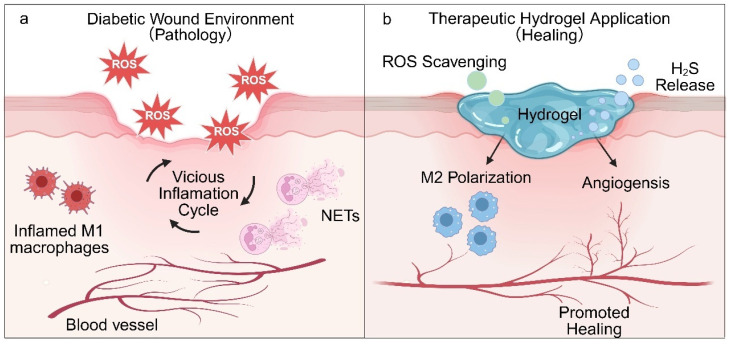
Oxidative stress–immune dysregulation cycle in diabetic CWs. (**a**) Excessive ROS sustains chronic inflammation with M1 macrophage activation and NETs formation. (**b**) ROS-responsive hydrogels scavenge ROS, promote M2 polarization and angiogenesis, facilitating wound healing. Created in BioRender. Huang, Q. (2026). BioRender.com/bhc3den.

Hyperglycemic conditions drive excessive production of ROS through AGEs accumulation and local hypoxia, while simultaneously suppressing endogenous antioxidant defenses, including superoxide dismutase (SOD) and glutathione peroxidase (GSH-Px) [69,70]. This results in a profound imbalance in redox homeostasis. Excessive ROS not only directly damages cellular structures and impairs cellular function but also activates inflammatory signaling pathways, such as NF-κB and MAPK, thereby promoting pro-inflammatory cytokine expression and suppressing anti-inflammatory mediators [71]. This redox imbalance directly interferes with macrophage polarization toward the M2 phenotype, delays neutrophil apoptosis, and enhances NETs formation, ultimately disrupting Treg cell function and T cell subset equilibrium. In turn, persistently activated inflammatory cells—including M1 macrophages and neutrophils—generate additional ROS via respiratory burst, establishing a self-reinforcing vicious cycle of “oxidative stress–immune dysregulation”. Concurrently, oxidative stress impairs endothelial function, inhibits angiogenesis, and obstructs wound revascularization, further delaying tissue repair [72,73]. To disrupt this pathological cycle, emerging therapeutic strategies have focused on oxidative stress-responsive biomaterials. For example, hydrogen sulfide (H_2_S)-releasing hydrogels designed to respond to elevated ROS levels represent a mechanism-driven intervention targeting the vicious cycle of oxidative stress and immune dysregulation, and can selectively release H_2_S within the wound microenvironment. This approach not only neutralizes excessive ROS but also promotes macrophage polarization toward the M2 phenotype, suppresses inflammation, and stimulates angiogenesis, thereby achieving coordinated regulation of redox balance and immune responses [74]. These findings underscore that the most promising therapeutic strategies involve smart responsive delivery systems—including ROS-sensitive hydrogels and nanocarriers—capable of dynamically sensing oxidative stress and spatiotemporally releasing antioxidants (e.g., H_2_S donors or Nrf2 activators) in combination with immunomodulatory agents. Such integrated platforms aim to restore redox equilibrium while simultaneously reshaping immune cell phenotypes, thereby offering mechanism-driven solutions for reversing chronic non-healing in diabetic wounds. The oxidative stress–immune dysregulation cycle and its therapeutic modulation are illustrated in Figure 4.

### 3.3. The Impact of Abnormal ECM Remodeling on the Immune Microenvironment

**Figure 5:**
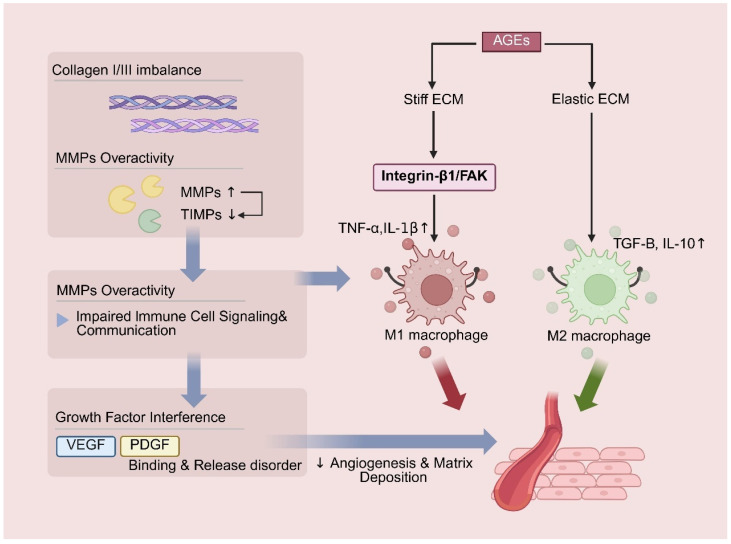
Abnormal ECM remodeling in diabetic CWs, characterized by collagen I/III imbalance and MMPs overactivity, alters integrin-β1/FAK-mediated macrophage polarization and disrupts growth factor signaling, thereby impairing angiogenesis and matrix deposition. Upward arrows (↑) indicate increased expression or activity, while downward arrows (↓) indicate suppression. Solid arrows represent activation or promotion of signaling pathways. Red-colored elements denote pro-inflammatory responses or M1 macrophage activity, whereas green-colored elements indicate anti-inflammatory responses or M2 macrophage activity. Blue arrows illustrate sequential pathological progression. Created in BioRender. Huang, Q. (2026). BioRender.com/5qjjxfc.

Abnormal ECM remodeling in diabetic wounds is primarily characterized by an imbalanced collagen I/III ratio and excessive degradation mediated by MMPs. Insufficient ECM synthesis combined with dysregulated degradation weakens the structural and biochemical framework required for immune cell recruitment and disrupts intercellular communication [75,76]. More importantly, AGE-mediated ECM stiffening exerts direct mechanobiological effects on immune cell behavior. A rigid ECM activates the integrin β1/FAK signaling pathway, thereby promoting macrophage polarization toward the pro-inflammatory M1 phenotype and enhancing secretion of mediators such as TNF-α and IL-1β. In contrast, an ECM with appropriate elasticity facilitates macrophage transition toward the reparative M2 phenotype [77,78]. Additionally, ECM abnormalities interfere with the binding, sequestration, and controlled release of growth factors such as VEGF and PDGF, further impairing angiogenesis and matrix deposition [79,80]. These mechanistic insights indicate that therapeutic strategies for diabetic wounds must move beyond passive structural ECM repair and instead actively modulate ECM-derived mechanical and biochemical cues to reshape the immune microenvironment. For instance, hydrogel scaffolds with dynamically tunable mechanical properties have been developed to adapt their stiffness across different healing stages, thereby guiding macrophage polarization toward a reparative phenotype [81]. Alternatively, MMP-responsive nanoparticles can be designed to selectively release tissue inhibitor of metalloproteinase (TIMP) mimetics in response to local MMP overexpression, while simultaneously delivering anti-fibrotic and pro-angiogenic factors. Through the integration of materials science and immunoregulation, such strategies aim to disrupt the vicious cycle of “ECM dysfunction–immune dysregulation–repair arrest,” offering next-generation therapeutic solutions for diabetic CWs that combine mechanistic depth with strong translational potential. The pathological mechanisms and immunoregulatory implications of abnormal ECM remodeling are illustrated in Figure 5.

## 4. Application of Immune-Regulating Smart Composite Nanocarriers

**Figure 6:**
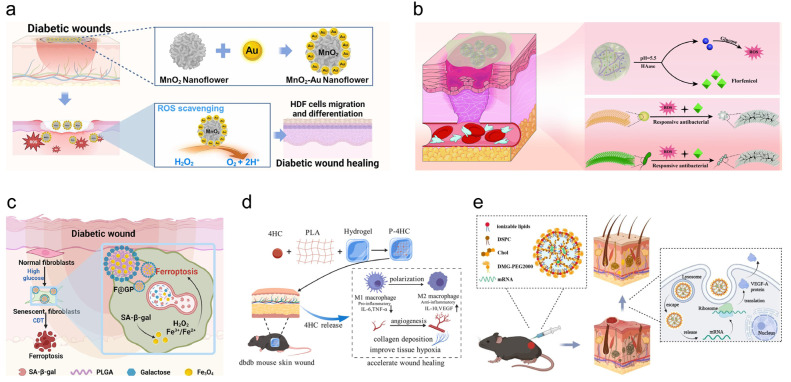
Application of Immune-Regulating Smart Composite Nanocarriers. (**a**) MnO_2_-Au nanoflowers efficiently scavenge ROS to accelerate diabetic wound healing. Reprinted with permission from Ref. [82]. (**b**) pH/HAase dual-responsive nanogels release florfenicol in response to the acidic environment and HAase in diabetic wounds. Reprinted with permission from Ref. [86]. (**c**) Fe_3_O_4_@Gal-PLGA nanospheres selectively target senescent fibroblasts in diabetic wounds by releasing iron ions in response to elevated SA-β-gal activity. Reprinted with permission from Ref. [91]. Copy right 2023, Elsevier. (**d**) The 4HC-loaded nanofiber hydrogel dressing modulates macrophage polarization from pro-inflammatory M1 to anti-inflammatory M2, enhancing angiogenesis, collagen deposition, and tissue oxygenation to accelerate diabetic wound healing. Reprinted with permission from Ref. [92]. Copy right 2025, Elsevier. (**e**) Ionizable lipid nanoparticles efficiently deliver VEGF-A mRNA to diabetic wounds, promoting its translation into VEGF-A protein, which stimulates angiogenesis and accelerates wound healing. Reprinted with permission from Ref. [96]. Copy right 2023, Elsevier. Arrows indicate activation, release, or directional progression of biological processes, and color variations represent different nanocarrier systems and corresponding biological responses.

Chronic non-healing diabetic wounds arise from a complex and dynamically evolving pathological microenvironment characterized by persistent inflammation, excessive oxidative stress, dysregulated immune cell behavior, impaired angiogenesis, aberrant ECM remodeling, and heightened susceptibility to infection. Conventional therapeutic approaches, which predominantly focus on single targets such as antimicrobial control or growth factor supplementation, have demonstrated limited efficacy in overcoming these multifactorial barriers, largely due to their inability to adapt to the spatiotemporal heterogeneity of the wound milieu. In this context, immune-regulating smart composite nanocarriers have emerged as a transformative therapeutic paradigm, integrating microenvironment-responsive material design with immunomodulatory and pro-regenerative functions. Rather than functioning as passive drug depots, these systems are engineered to sense pathological cues—such as elevated ROS, acidic pH, aberrant enzyme activity, metabolic dysregulation, and immune imbalance—and translate them into precisely coordinated biological interventions. By coupling stimulus-responsive release with active regulation of immune responses, redox homeostasis, vascular regeneration, and stromal remodeling, smart composite nanocarriers enable synchronized modulation across multiple phases of wound healing. Accordingly, this section systematically summarizes recent advances in immune-regulating smart composite nanocarriers, with a particular focus on ROS-responsive, pH-responsive, enzyme-responsive, macrophage polarization–regulating, growth factor–immune modulator co-loaded, and stem cell-based composite systems, highlighting their design principles, mechanistic insights, and therapeutic potential for achieving durable and high-quality diabetic wound repair. Representative immune-regulating smart composite nanocarriers and their mechanisms are illustrated in Figure 6.

### 4.1. Reactive Oxygen Species-Responsive Carriers

Excessive and persistent accumulation of ROS represents a central pathological hallmark of diabetic wounds, driving sustained inflammation, ECM degradation, impaired angiogenesis, and increased susceptibility to infection. Importantly, ROS in diabetic wounds are not transient byproducts but chronically elevated signals generated by hyperglycemia-induced mitochondrial dysfunction, AGEs–RAGE activation, and persistent inflammatory cell infiltration. Sustained ROS accumulation stabilizes NF-κB signaling, maintains macrophages in a pro-inflammatory M1 state, promotes NETs formation, and suppresses endothelial nitric oxide bioavailability, thereby mechanistically linking oxidative stress to immune dysregulation and vascular dysfunction. In this context, ROS-responsive nanocarriers offer a distinct therapeutic advantage by converting pathological redox imbalance into a site-specific activation signal, enabling selective drug release or catalytic activity exclusively within oxidative stress-enriched wound regions. For example, nanozyme-based systems such as manganese dioxide nanoflower hybridized gold nanoparticle composites (MnO_2_-Au nanoflowers) exhibit catalase- and peroxidase-like activities, decomposing excess hydrogen peroxide (H_2_O_2_) into oxygen and water, which simultaneously reduces oxidative damage and alleviates local hypoxia. This dual effect directly interrupts ROS-driven inflammatory amplification while restoring oxygen-dependent angiogenic and fibroblast functions [82]. Beyond inorganic nanozymes, ROS-responsive metal–organic framework-based systems, exemplified by BR@Zn-BTB nanoparticles embedded in antioxidant hydrogels, integrate redox-triggered structural degradation with controlled release of bioactive ions and small molecules. By synchronizing ROS scavenging with Zn^2+^-mediated anti-inflammatory signaling and suppression of MMP-9 overactivation, these platforms directly target ECM destabilization and inflammatory persistence, rather than merely accelerating wound closure [83]. Notably, emerging ROS-responsive hydrogels and composite systems increasingly incorporate additional functional modules, including antibacterial photothermal agents, angiogenic cues, and glucose-regulating components. Such designs reflect a mechanistic shift from single-endpoint antioxidant therapy toward redox-guided, multiphase intervention strategies that address infection control, immune resolution, and matrix reconstruction in a coordinated manner [84,85].

Collectively, ROS-responsive nanocarriers should not be viewed merely as passive antioxidant depots but as redox-activated regulatory systems capable of coupling pathological ROS gradients to localized therapeutic activation. However, it is increasingly evident that ROS scavenging alone is insufficient to fully reprogram the chronic inflammatory and reparative arrest characteristic of diabetic wounds. Excessive attenuation of ROS may inadvertently impair redox-dependent physiological processes, including antimicrobial defense and angiogenic signaling, underscoring the need for quantitatively controlled rather than indiscriminate ROS elimination.

From a mechanistic perspective, the true value of ROS-responsive systems lies not in maximizing antioxidant capacity, but in using ROS as a contextual trigger to synchronize immunomodulation, vascular restoration, and ECM stabilization with disease stage and microenvironmental demand. Future designs must therefore prioritize threshold-sensitive responsiveness, integration with complementary pathological cues (e.g., pH, protease activity, or metabolic stress), and functional coupling to downstream immune and stromal pathways, rather than continued escalation of ROS-scavenging efficiency. Such considerations are critical for translating ROS-responsive nanocarriers from proof-of-concept antioxidant platforms into clinically relevant, stage-adaptive regulators of diabetic wound repair.

### 4.2. pH-Responsive Carriers

The diabetic wound microenvironment is characterized by pronounced pH dysregulation arising from bacterial metabolism, sustained inflammation, hypoxia, and aberrant glucose oxidation, rendering local acidity a robust and disease-specific stimulus for smart therapeutic intervention. pH-responsive carriers, especially in the form of hydrogels and nanogels, have emerged as a promising solution due to their ability to deliver drugs in response to the fluctuating pH at the wound site. Recent advancements have shown that the incorporation of glucose oxidase (GOx) facilitates the breakdown of glucose into gluconic acid and H_2_O_2_, lowering the wound’s pH and generating ROS [86]. These ROS not only disrupt bacterial biofilms, which are notoriously difficult to treat, but also contribute to wound healing by reducing oxidative stress, modulating inflammation, and promoting angiogenesis. An example of this approach is the development of N, O-carboxymethyl chitosan (N, O-CMCS) and oxidized hyaluronic acid (OHA)-based nanogels, which release glucose oxidase on demand in response to the acidic conditions of diabetic wounds, thus optimizing therapeutic outcomes [87].

Despite promising results, several challenges remain in maximizing the clinical potential of pH-responsive carriers. The dual response of these systems, especially those sensitive to both pH and glucose, enhances efficacy but also raises concerns about consistency and predictability. For example, Fe-doped ZIF-8 nanozymes lower glucose levels and catalyze ROS production, addressing bacterial infections and oxidative stress [88]. However, the reliability of these responses may vary in patients with fluctuating glucose levels or differing wound conditions. Additionally, the complex interactions between GOx, ROS, and nanomaterials may cause unpredictable effects on surrounding tissues, requiring better control over drug release and enzymatic activity.

To overcome these challenges, future research should focus on developing robust formulations with improved predictability and consistency across patients and environments. More adaptive drug release mechanisms, capable of responding to specific wound conditions, are crucial for optimizing therapeutic benefits. Furthermore, addressing issues related to scalability and ensuring long-term safety through comprehensive preclinical and clinical studies are key steps for making these systems viable for widespread clinical use. With these advancements, pH-responsive carriers have the potential to offer a synergistic solution to the multifaceted challenges of diabetic wound healing, delivering sustained therapeutic effects across diverse patient populations.

### 4.3. Enzyme-Responsive Nanocarriers

Enzyme-responsive nanocarriers have emerged as a powerful strategy for diabetic wound healing by exploiting the pathological overexpression of endogenous enzymes to achieve site-specific drug activation, retention, and therapeutic amplification. In diabetic wounds, MMPs, particularly MMP-2 and MMP-9, are markedly upregulated as a consequence of sustained inflammation and ECM dysregulation. This pathological feature has been leveraged to design enzyme-responsive delivery systems that remain inert under physiological conditions but undergo structural transformation or drug release upon enzymatic cleavage in diseased tissues. For example, hyaluronic acid end-conjugated polyamidoamine dendrimers incorporating MMP-2-cleavable peptide linkers enable selective release of antioxidant astragaloside at diabetic wound sites, thereby enhancing redox balance, promoting keratinocyte and fibroblast migration, and accelerating tissue regeneration [89]. Similarly, peptide–polymer amphiphile nanoparticles responsive to MMP activity have been shown to undergo enzyme-triggered morphological transitions from nanoscale assemblies to microscale aggregates, resulting in prolonged retention of dexamethasone within inflamed diabetic tissues and significantly improved local anti-inflammatory efficacy compared with free drug administration [90]. Beyond inflammation modulation, recent advances have extended enzyme-responsive strategies toward targeting upstream pathological drivers of chronic wounds, such as cellular senescence. Nanospheres engineered to respond to senescence-associated β-galactosidase selectively release catalytic components that induce chemodynamic therapy within senescent cells, leading to their ferroptotic elimination, suppression of senescence-associated secretory phenotypes, and restoration of a pro-regenerative wound microenvironment [91].

Taken together, current evidence indicates that enzyme-responsive nanocarriers enable precise spatiotemporal control over therapeutic activation within diabetic wound microenvironments by exploiting disease-associated enzymatic cues, thereby confining drug release, structural transformation, or catalytic activation to sites of pathological relevance. Beyond enhancing local drug bioavailability and retention, such systems function as integrative therapeutic platforms that simultaneously modulate multiple interconnected pathological processes, including the resolution of chronic inflammation, restoration of redox homeostasis, regulation of ECM turnover, and selective elimination of detrimental cell populations such as senescent cells. By aligning material responsiveness with endogenous enzymatic signatures of diabetic wounds, enzyme-responsive nanocarriers shift treatment paradigms from passive drug delivery toward mechanism-driven, microenvironment-adaptive interventions that support high-quality tissue regeneration.

### 4.4. Macrophage Polarization-Regulating Nanocarriers

Dysregulated macrophage polarization constitutes a central immunopathological barrier to effective diabetic wound healing. In diabetic wounds, prolonged dominance of the pro-inflammatory M1 phenotype perpetuates cytokine overproduction, oxidative stress, and protease-mediated ECM degradation, whereas impaired transition toward the reparative M2 phenotype compromises angiogenesis, fibroblast activation, and orderly tissue remodeling. Accumulating evidence indicates that targeted modulation of macrophage polarization can reprogram the wound immune microenvironment and restore regenerative progression. At the molecular level, small molecules such as resveratrol promote M2 polarization via activation of the PI3K/Akt signaling pathway, thereby attenuating chronic inflammation and enhancing collagen deposition [53]. Building upon these biological insights, biomaterial-based strategies further amplify immunomodulatory efficacy by enabling localized, sustained, and microenvironment-adaptive regulation of macrophage phenotypes. For example, nanofiber hydrogel dressings loaded with 4′-hydroxychalcone (4HC) dynamically suppress TLR-, IL-17-, and TNF-related inflammatory signaling, thereby driving macrophage reprogramming toward an M2-dominant state while concurrently alleviating oxidative stress and enhancing angiogenesis [92]. In parallel, multi-responsive hydrogels combined with mild thermal stimulation synergistically promote M2 polarization and vascular maturation by integrating inflammatory regulation with pro-angiogenic cues [93]. Beyond synthetic systems, lean adipose tissue macrophage-derived exosomes provide compelling biological evidence that macrophage fate can be remotely regulated through microRNA-mediated signaling, exemplified by miR-222-3p-dependent suppression of Bim, which facilitates M1-to-M2 transition and accelerates diabetic wound repair [94].

Precise control of macrophage polarization represents a fundamental regulatory axis in diabetic wound healing rather than a passive downstream consequence. By orchestrating the timely transition from pro-inflammatory to reparative immune states, macrophage polarization synchronizes inflammation resolution, redox homeostasis, angiogenesis, fibroblast activity, and ECM remodeling across distinct healing phases. Consequently, therapeutic strategies that actively target macrophage polarization shift the treatment paradigm from symptomatic wound coverage toward mechanism-driven regeneration, positioning immunomodulation as a cornerstone of next-generation diabetic wound therapies.

### 4.5. Growth Factor-Immune Modulator Co-Loaded Nanocarriers

While targeted regulation of macrophage polarization has been recognized as a central mechanism for resolving chronic inflammation and restoring regenerative progression in diabetic wounds, immune reprogramming alone is often insufficient to fully reconstruct the severely compromised vascular and stromal compartments. In the diabetic wound microenvironment, persistent hypoxia, endothelial dysfunction, and attenuated growth factor signaling coexist with immune dysregulation, necessitating therapeutic strategies that extend beyond single-axis immunomodulation. Accordingly, recent advances have shifted toward integrative nanocarrier-based platforms that couple immune regulation with direct pro-regenerative cues, particularly angiogenic growth factors, to synchronize inflammatory resolution with vascular reconstruction and tissue remodeling. For instance, bioactive dermal matrix-based hydrogels incorporating VEGF- and IL-10-loaded microspheres enable sustained and localized release of angiogenic and anti-inflammatory cues, simultaneously enhancing neovascularization and suppressing chronic inflammation, which collectively accelerates wound closure and improves tissue mechanical integrity [95]. Beyond protein-based approaches, nucleic acid-based strategies have emerged as powerful alternatives, as exemplified by ionizable lipid nanoparticles delivering VEGF-A mRNA to achieve transient yet robust in situ protein expression, resulting in pronounced enhancement of endothelial cell proliferation, vascular density, and re-epithelialization in diabetic wounds [96]. Importantly, combinatorial mRNA strategies further extend this concept by demonstrating that co-delivery of VEGF-A and stabilized FGF1 mRNAs produces additive or synergistic effects on angiogenesis and tissue regeneration, outperforming single-factor mRNA therapies in both ex vivo and in vivo models [97]. Complementing these approaches, hydrogel-mediated delivery of lipid nanoparticle-formulated PTD-BMP2 enables efficient intracellular transduction and prolonged local bioactivity of BMP2, thereby promoting not only angiogenesis but also vascular maturation and ECM remodeling while minimizing dose-related adverse effects [98].

Growth factor–immunomodulator co-delivery systems establish a mechanistically coherent therapeutic framework that bridges immune reprogramming with structural regeneration in diabetic wounds. By simultaneously attenuating persistent inflammation, reshaping macrophage polarization, and restoring angiogenic and stromal signaling, these integrated platforms overcome the intrinsic limitations of monotherapies that target isolated pathological features. Importantly, the spatiotemporally coordinated presentation of immunomodulatory cues and pro-regenerative growth factors enables precise synchronization of inflammatory resolution with vascular reconstruction, fibroblast activation, and ECM remodeling. As a result, such multifunctional nanocarriers not only accelerate wound closure but also enhance tissue maturation and mechanical integrity, thereby promoting durable and high-quality repair. Taken together, these advances position growth factor–immunomodulator co-delivery strategies as a foundational paradigm for next-generation diabetic wound therapeutics, shifting the treatment focus from transient symptom alleviation toward mechanism-driven microenvironmental restoration.

### 4.6. Stem Cell-Nanocarrier Composite System

Stem cell-based therapies have emerged as a promising approach for diabetic wound repair due to their potent paracrine and immunomodulatory effects; however, their clinical efficacy is often limited by poor cell survival, rapid loss from the wound site, and insufficient control over the hostile diabetic microenvironment. To overcome these barriers, recent studies have increasingly focused on integrating stem cells or their secretome with advanced nanocarrier or hydrogel delivery platforms to enhance therapeutic durability and precision. For example, encapsulation of Wharton’s jelly-derived mesenchymal stem cells (MSCs) within hydrogel matrices markedly improves cell retention and viability in diabetic wounds, enabling sustained paracrine signaling that promotes macrophage polarization toward a reparative M2 phenotype, enhances angiogenesis, and drives antifibrotic ECM remodeling characterized by an increased collagen III/I ratio [99]. Complementing cell-based delivery, multifunctional hydrogels engineered to deliver mesenchymal stem cell secretome provide a cell-free yet highly effective alternative, as exemplified by ROS- and glucose-responsive systems that dynamically release bioactive factors to suppress excessive NETs formation via the PGE2/BMAL1 axis, thereby alleviating inflammatory damage and restoring a regenerative immune microenvironment [100]. In parallel, stem cell factor-loaded collagen-based hydrogels further demonstrate that controlled presentation of stem cell-derived bioactive cues can simultaneously enhance fibroblast activity, promote angiogenesis, and modulate macrophage polarization, even in the absence of transplanted cells [101].

Stem cell–nanocarrier composite systems operate not merely as passive delivery vehicles for cells or bioactive factors, but as active and adaptive regulators of the diabetic wound microenvironment. By enhancing stem cell retention and viability or enabling sustained and stimulus-responsive release of stem cell-derived secretome components, these platforms effectively counteract the hostile conditions characteristic of diabetic wounds, including excessive inflammation, oxidative stress, and protease-mediated ECM degradation. Importantly, the integrated modulation of immune responses, exemplified by suppression of NETs formation and promotion of macrophage polarization toward a reparative M2 phenotype, is tightly coupled with restoration of angiogenic signaling, fibroblast activation, and ECM remodeling. Through this coordinated regulation across inflammatory, vascular, and stromal compartments, stem cell–nanocarrier composite systems synchronize immune resolution with structural regeneration, thereby not only accelerating wound closure but also improving tissue maturity, mechanical integrity, and antifibrotic healing outcomes. Together, these advances position stem cell–nanocarrier-based strategies as a robust and translatable therapeutic paradigm for achieving durable and high-quality diabetic wound repair, moving beyond short-lived symptomatic improvement toward mechanism-driven microenvironmental restoration. Representative immune-regulating smart composite nanocarriers and their corresponding pathological triggers are further summarized in Table 2.

## 5. Application of Artificial Intelligence in Wound Monitoring and Treatment

With the rapid advancement of smart technologies in the medical field, AI and machine learning have demonstrated substantial potential in wound monitoring and therapeutic optimization. Traditional clinical monitoring approaches for diabetic foot ulcers (DFUs) suffer from significant limitations, including subjectivity, delayed feedback, and limited capacity for personalized intervention. These constraints hinder timely decision-making and fail to capture the dynamic and heterogeneous nature of DFU healing. In contrast, AI-driven technologies enable continuous, quantitative, and data-driven assessment, offering novel opportunities for dynamic wound management and individualized treatment optimization. Machine learning algorithms—such as k-nearest neighbors (KNN), artificial neural networks (ANN), and convolutional neural networks (CNN)—have been increasingly applied in DFU care, demonstrating distinct advantages in real-time monitoring, therapeutic prediction, and risk stratification. Accordingly, this section summarizes how different AI paradigms support complementary aspects of DFU management, ranging from wound state monitoring and outcome prediction to drug-delivery optimization and sensor-integrated smart dressing systems, with an emphasis on their underlying principles, methodological strengths, and representative clinical or preclinical use cases.

### 5.1. KNN for Real-Time Wound Monitoring and Early Complication Detection

The KNN algorithm, as a “lazy learning” classification approach, exhibits high efficiency in real-time analytical tasks, particularly when processing low-dimensional physiological sensor data such as glucose concentration and pH, which are critical parameters in diabetic wound monitoring [110]. KNN operates on the principle of local similarity, classifying incoming data points by calculating Euclidean distances to neighboring samples within the training dataset. Typically, k values ranging from 3 to 7 are selected to balance classification accuracy and noise tolerance. A key advantage of KNN lies in its minimal computational latency and absence of pre-training requirements, enabling flexible adaptation to rapid microenvironmental fluctuations at the wound site.

In practical applications, KNN has been successfully integrated into advanced sensing platforms to enhance real-time decision-making in diabetic wound care. For example, a stretchable bioelectronic dressing incorporating KNN with PEDOT: PSS-based sensors achieved 92% accuracy, 89% sensitivity, and 95% specificity across 500 clinical DFU samples [110]. This system enabled early infection alerts within two hours of bacterial inoculation—approximately 24-fold faster than conventional clinical observation—and significantly reduced bacterial burden from 10^7^ CFU·g^−1^ to 10^4^ CFU·g^−1^. Beyond infection detection, KNN has also been applied to monitor mechanical strain at wound sites to prevent secondary injury. Zhang et al. integrated KNN into an ion-based hydrogel system with ROS-scavenging capability for real-time strain classification, achieving 89% accuracy in diabetic rat models and effectively preventing delayed healing caused by abnormal mechanical stress [111].

Moreover, KNN has demonstrated utility in multidimensional physiological signal analysis. When combined with ROS-sensitive nanofiber dressings, KNN-enabled systems can dynamically assess oxidative stress levels and regulate silver nanoparticle release accordingly, resulting in significant bacterial load reduction in DFUs [112]. Collectively, these studies highlight that the core value of KNN lies in its role as a lightweight, adaptive real-time signal analysis engine, capable of seamless integration with smart materials to construct closed-loop wound management systems encompassing “sensing–analysis–intervention.” Future advances will depend on improving KNN robustness for higher-dimensional, nonlinear physiological datasets and enhancing its compatibility with edge computing architectures and autonomous therapeutic actuators.

### 5.2. ANN for Optimizing Wound Treatment and Drug Release

Artificial neural networks (ANNs) have emerged as powerful tools for modeling the complex, nonlinear dynamics of diabetic wound healing owing to their strong capability for multivariate data integration and nonlinear optimization. By incorporating multidimensional inputs—including dressing material properties, electrical stimulation parameters, and biochemical microenvironmental variables such as glucose and pH—ANNs can accurately predict key healing outcomes, including vascular density, drug release kinetics, and epithelialization rates, while enabling iterative optimization through backpropagation algorithms [113].

In practical applications, ANN-based models have demonstrated significant value in precision optimization of therapeutic parameters. For instance, ANN-guided optimization of electrical stimulation conditions identified an optimal field strength of 100 mV·mm^−1^ applied for 1 h, resulting in a marked increase in CD31^+^ vascular density in diabetic rat wounds from 25 to 57.5 vessels·mm^−2^ [114]. This example illustrates the capacity of ANNs to fine-tune complex physical interventions in a data-driven manner. Similarly, ANNs have been applied to optimize metformin release profiles from glucose- and pH-responsive hydrogels, enabling stage-specific drug delivery: rapid release of approximately 80% of the drug within the first 6 h to control acute inflammation, followed by sustained release of 40% during the subsequent repair phase to avoid excessive immunosuppression. This strategy increased wound closure rates in diabetic rats from 35% to 82% within 14 days [115].

Overall, the central value of ANN in diabetic wound management lies in its ability to establish closed-loop intelligent decision-making systems, linking multimodal sensing inputs with personalized therapeutic outputs. Future development should prioritize lightweight ANN architectures deployable on edge devices, enabling real-time processing of wound sensor data and dynamic adjustment of therapeutic parameters such as electrical stimulation, drug release rates, or bioactive factor delivery. Through deep integration with Internet-of-Things (IoT) platforms and automated smart dressings, ANN-driven systems have the potential to transform diabetic wound care from static, experience-based treatment into continuously optimized precision therapy.

### 5.3. CNN for Early Lesion Detection and Wound Healing Evaluation

Convolutional neural networks (CNNs) have become a cornerstone technology for objective and automated analysis of diabetic wound images, owing to their powerful spatial feature extraction capabilities. Through hierarchical convolutional and pooling operations, CNNs enable automated segmentation of wound regions and precise quantification of tissue components, such as granulation tissue and necrotic tissue, thereby providing reproducible and quantitative metrics for lesion assessment and healing evaluation.

In early lesion detection, CNN models based on architectures such as MobileNetV2 have been developed for the identification of early maceration in DFUs. Trained on 1200 clinical images, these models achieved 89% classification accuracy, outperforming board-certified dermatologists and reducing identification time from 48 h to approximately 6 h, thereby enabling timely early intervention [116]. For tissue composition analysis, U-Net-based architectures have been widely employed to perform fine-grained segmentation of DFUs, effectively distinguishing necrotic from granulation tissue and eliminating subjectivity inherent in manual assessment [117]. Importantly, CNN-based image analysis has been increasingly integrated with smart material platforms. For example, embedding CNN algorithms within transparent conductive hydrogels enables real-time, noninvasive monitoring of epithelialization dynamics, with measurement errors below 5%, and automatically triggers intervention alerts during healing stagnation [118]. CNNs have also demonstrated strong predictive capability in risk stratification. Hybrid ANN–CNN models combining CNN-segmented necrotic tissue ratios with clinical parameters have achieved 91% accuracy in predicting 6-month amputation risk, significantly enhancing clinical decision-making and early intervention planning [119].

**Figure 7:**
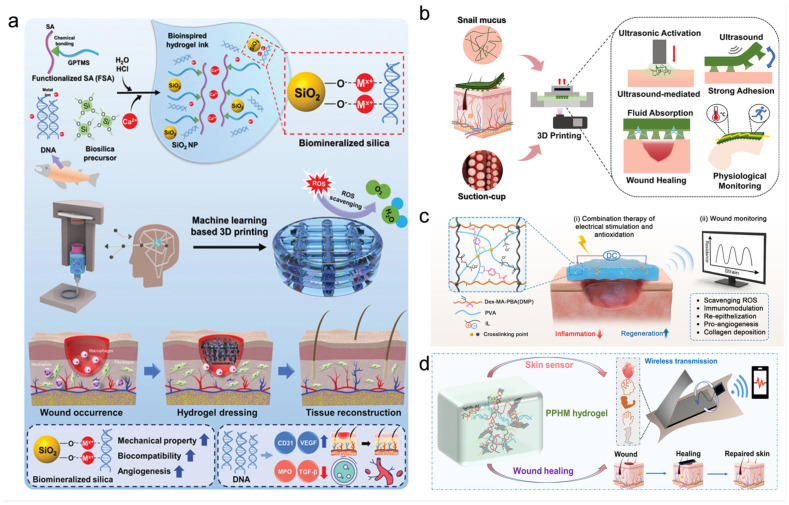
Application of AI in Wound Monitoring and Treatment. (**a**) The 3D-printed biomineralized silica hydrogel enhances diabetic wound healing by promoting macrophage migration, angiogenesis, and collagen deposition through controlled release of DNA and growth factors. Reprinted with permission from Ref. [120]. Copy right 2024, Elsevier. (**b**) 3D-printed bioinspired hydrogel patch with enhanced adhesion, fluid absorption, and ultrasound-activated monitoring for accelerated wound healing. Reprinted with permission from Ref. [121]. Copy right 2024, Elsevier. (**c**) A conductive multifunctional hydrogel combines ROS-scavenging and electrical stimulation to treat chronic diabetic wounds, reduce inflammation, and enhance tissue regeneration, while also serving as a strain sensor for real-time wound monitoring. Reprinted with permission from Ref. [111]. (**d**) The PPHM hydrogel sensor enables real-time monitoring of wound healing and accelerates the healing process by promoting proliferation, migration, and angiogenesis in wounds. Reprinted with permission from Ref. [122]. Copy right 2023, Elsevier. Arrows indicate activation, release, or directional progression of therapeutic and monitoring processes, and color variations represent different biomaterials, biological responses, or functional components within each system.

Collectively, the significance of CNNs in diabetic wound management extends beyond image automation. Their broader impact lies in enabling three paradigm shifts: (i) transition from subjective visual assessment to objective quantitative evaluation; (ii) evolution from static snapshot analysis to continuous dynamic monitoring; and (iii) progression from reactive complication management to proactive risk prediction and personalized prevention. With further development of lightweight CNN models suitable for bedside or wearable deployment and integration with electronic health records and multi-omics datasets, CNNs are poised to serve as core analytical engines driving precision wound care. The integrated AI-driven sensing and predictive framework described above is illustrated in Figure 7.

### 5.4. Application of Nanoinformatics and Computational Models in Nanocarrier Design

Nanoinformatics provides a data-driven framework for rationalizing nanocarrier design beyond empirical trial-and-error approaches, which is particularly relevant for therapeutic systems operating in complex biological environments. By integrating multidimensional descriptors—including material composition, surface chemistry, formulation parameters, and biological responses—nanoinformatics enables predictive modeling of nanocarrier performance across multiple design levels.

Recent comprehensive studies have demonstrated that machine learning-based nanoinformatics models can accurately predict key formulation attributes, such as particle size distribution, drug loading efficiency, biodistribution, and toxicity profiles, by capturing nonlinear relationships between synthesis variables and biological outcomes [123]. These approaches substantially reduce experimental burden and improve early-stage decision-making by prioritizing candidates with favorable physicochemical and safety profiles. In parallel, AI-assisted formulation platforms and curated nanoinformatics databases have further enhanced reproducibility and cross-study comparability, providing standardized data infrastructures for predictive modeling and translational evaluation. Collectively, these advances position nanoinformatics as a powerful enabling technology for accelerating nanocarrier development and improving translational efficiency [81].

However, from a disease-oriented perspective, many current nanoinformatics models remain primarily focused on optimizing generic material properties rather than capturing the dynamic biological demands of chronic wound repair. In diabetic wounds, therapeutic efficacy is determined not only by carrier stability or delivery efficiency, but also by the ability to interact with evolving inflammatory, metabolic, and proteolytic microenvironments. This consideration underscores the necessity of advancing nanoinformatics toward function-oriented modeling frameworks that align computational design with immunomodulatory outcomes and microenvironment-adaptive therapeutic responses, thereby enhancing the biological relevance and translational value of AI-guided nanocarrier development.

### 5.5. Optimization of Drug Release via Predictive Models and Smart Carriers

In the treatment of diabetic CWs, optimizing drug release is crucial due to the challenges posed by chronic inflammation, immune dysregulation, and poor local circulation. The formation of the protein corona, a layer of proteins that spontaneously adsorbs onto the surface of nanoparticles when they interact with biological fluids, significantly impacts nanoparticle biocompatibility, immune responses, and drug release stability. Predicting the composition and dynamic changes of the protein corona is key to optimizing drug release, ensuring that drugs are delivered accurately and effectively to the target site.

For instance, a study involving gold nanoparticles exposed to serum demonstrated how different surface properties directly influence the composition of the protein corona. The composition of this protein corona, in turn, affects nanoparticle stability, immune responses, and drug release characteristics. This finding underscores the importance of predicting protein corona formation, as it not only improves the stability of nanoparticles but also enhances targeting and optimizes drug release properties. By integrating machine learning models, researchers can predict how protein corona formation impacts drug delivery, enabling the design of more efficient drug delivery systems [124]. Furthermore, the dynamic nature of the protein corona also plays a critical role in drug release optimization. When DNA nanostructures are exposed to serum, they adsorb specific proteins, forming distinct protein coronas. By functionalizing DNA nanostructures with modifications such as cholesterol or polylysine, the composition of the protein corona can be regulated, which influences drug release properties. Cholesterol-functionalized DNA nanostructures (Th-Ch) form enriched protein coronas, which enhance the stability and targeting ability of the nanoparticles, allowing for more precise drug release at the wound site. This prediction-based strategy for optimizing drug release through protein corona formation has shown significant potential in treating diabetic CWs [125].

Despite significant progress, challenges remain in protein corona prediction and optimization for drug release. The diabetic wound environment is complex, and the dynamic nature of the protein corona is influenced by factors such as oxidative stress, immune responses, and local pH fluctuations. Additionally, individual variability means that the protein corona composition may differ significantly between patients, complicating predictions. Future research should focus on incorporating real-time wound environment data and multi-omics information to improve the accuracy of protein corona predictions and optimize drug release strategies

### 5.6. Real-Time Analysis of Smart Dressings Integrated with Sensors

The integration of sensors into wound dressings has revolutionized wound care, particularly for chronic and diabetic wounds that require continuous monitoring and intervention. Traditional wound management methods, based on periodic assessments or ex situ testing, fall short in providing real-time insights into the dynamic wound microenvironment. However, smart wound dressings allow for continuous, in situ monitoring, enabling clinicians to make more informed decisions and personalize treatments.

A key example is the smart hydrogel dressing (GelDerm), which integrates pH and glucose sensors alongside drug delivery systems. The pH sensor detects changes in the wound’s acidity, indicating potential infection, while the glucose sensor continuously monitors glucose levels, a crucial factor in diabetic wound healing. This dual functionality enables real-time assessment and active intervention, as the dressing also delivers antibiotics and growth factors to support healing. This approach accelerates wound closure and improves healing outcomes, showcasing the potential of sensor-integrated dressings as multifunctional therapeutic platforms rather than purely diagnostic tools [126]. Similarly, another advancement comes from a sprayable hydrogel system, which incorporates optical mRNA nanosensors (NanoFlares) for real-time monitoring of gene expression related to inflammation and tissue remodeling. By targeting specific mRNAs such as PECAM1 and COL1A1, this system provides a more detailed molecular-level understanding of wound healing. The hydrogel also delivers LL37, an antimicrobial peptide, adding antimicrobial and pro-healing properties to the dressing. This integration of sensing and therapy represents a significant leap forward, as it allows for the direct modulation of the wound environment based on real-time molecular data [127].

Most existing systems rely on a limited set of biomarkers, which may not fully capture the complexity of chronic wound healing. Moreover, sensor accuracy can be affected by factors such as temperature, storage conditions, and wound exudate composition, which may undermine their performance in real-world applications. Meanwhile, the drug delivery systems in many smart dressings still operate based on preset algorithms rather than real-time sensor feedback, limiting their ability to adapt dynamically to the wound’s changing needs. Future research should focus on the development of next-generation smart wound dressings that integrate multimodal sensing with responsive drug delivery systems. Expanding the range of biomarkers and incorporating real-time adaptive mechanisms would significantly improve wound monitoring and therapy. Furthermore, the integration of AI and machine learning for data analysis and predictive modeling will play a crucial role in advancing these technologies toward personalized and stage-aware wound care. Such innovations could transform wound management, offering patients more efficient, accurate, and individualized treatments.

## 6. Clinical Progress and Limitations of Smart Composite Nanocarriers in Diabetic Wound Healing

Smart composite nanocarriers, such as silver nanoparticles (AgNPs) and zinc oxide nanoparticles (ZnO NPs), have shown significant promise in diabetic CWs treatment due to their unique properties, including antimicrobial activity, tissue regeneration, and modulation of inflammatory responses. Clinical trials have demonstrated that silver nanoparticle-based dressings (SilverSTAT Gel) significantly reduce healing times and improve wound closure rates [128]. In a randomized controlled trial, 90% of patients using SilverSTAT Gel achieved complete wound closure by the 12th week, compared to 77.5% in the control group. The effectiveness of silver nanoparticles is largely attributed to their ability to prevent infection, reduce inflammation, and promote tissue regeneration through enhanced fibroblast migration and angiogenesis [129]. Similarly, ZnO nanoparticle-infused calcium alginate dressings have also demonstrated faster wound healing and better closure rates compared to conventional treatments, showing a potential for enhancing antimicrobial efficacy and accelerating tissue repair [130].

Beyond published clinical outcomes, registered clinical trials provide additional insight into the current state of translational development. Notably, a completed interventional study registered on ClinicalTrials.gov (NCT04834245) evaluated a hydrogel/nano-silver-based dressing in patients with diabetic foot wounds, directly comparing a nano-silver composite dressing with conventional wound dressings [131]. This study represents one of the few clinically registered investigations explicitly assessing a nanomaterial-based smart dressing in diabetic wound care. Its design reflects the translational rationale of AgNP-based systems, in which a hydrogel carrier is employed to enhance local retention, sustain antimicrobial activity, and modulate the wound microenvironment.

In addition to nanoparticle-based systems, biologically derived therapies like a cell-free secretome from stressed peripheral blood mononuclear cells (APOSEC), are emerging as innovative treatments [132]. Preliminary data from the ongoing MARSYAS II trial suggest that APOSEC can significantly reduce wound size by modulating immune responses, enhancing angiogenesis, and promoting fibroblast and keratinocyte migration. A completed randomized trial (NCT05671250) assessed a bioactive smart dressing incorporating platelet-rich plasma (PRP) gel in diabetic foot ulcers, underscoring growing clinical interest in cell-free bioactive formulations capable of modulating inflammation and promoting tissue regeneration [133]. These findings emphasize the growing potential of biologically derived nanomaterials in improving wound healing, particularly in chronic diabetic ulcers resistant to traditional therapies.

Smart nanocarriers have demonstrated considerable potential in advancing diabetic wound healing, yet several critical challenges remain that must be addressed to facilitate their broader clinical implementation. Among the primary obstacles are the formation of the protein corona, rapid drug release kinetics, and the delicate balance between personalization and scalability. From a methodological perspective, appropriate statistical analysis—such as time-to-closure evaluation using survival-based approaches—remains important for robust assessment of therapeutic efficacy in clinical studies. In the complex environment of diabetic wounds, the exudate, rich in proteins and enzymes, forms a ‘protein corona’ around nanoparticles, which alters their surface properties and may compromise their therapeutic efficacy. Additionally, the fast release of therapeutic payloads by many nanocarriers is ill-suited for the chronic, slow-healing nature of diabetic wounds, underscoring the need for more refined systems capable of sustained, controlled, or responsive release. While personalized nanocarriers, such as those designed for miRNA-based therapies, hold the promise of precision medicine, their high production costs and intricate design present significant scalability challenges. Moreover, safety concerns, including adverse reactions to nanoparticles and their long-term effects on the skin microbiome and wound healing, further complicate their clinical use. Addressing these barriers is crucial for the successful translation of nanocarrier-based therapies into routine clinical practice, ensuring that their full potential in diabetic wound care can be realized.

## 7. Conclusions and Future Perspectives

Despite the promising potential of immune-regulating smart composite nanocarriers for diabetic CWs treatment, several critical barriers still hinder their clinical translation. Currently, most nanocarriers are designed based on a single stimulus response, such as ROS or pH, which fails to account for the asynchronous progression of wound healing in different stages. For example, the early inflammatory phase and the later regenerative phase demand distinct therapeutic interventions. As demonstrated in numerous studies, the dynamic changes in inflammation, oxidative stress, protease activity, and ECM remodeling determine the various phases of wound healing, meaning that a universal nanocarrier design based on single-response mechanisms is unable to meet the dynamically evolving needs of therapy. To improve the therapeutic efficacy, the design of nanocarriers should be flexible enough to respond to these changes in real time, providing stage-specific interventions, and future development should emphasize quantifiable and programmable multi-signal response logics that enable controlled, stage-matched therapeutic actions rather than simply intensifying a single stimulus sensitivity.

AI holds significant potential in guiding the treatment of diabetic CWs by integrating clinical and biological data to predict healing risks and optimize treatment strategies. However, current AI applications are limited in their capabilities. Most AI models primarily rely on imaging data or a narrow set of physiological parameters, such as glucose levels and wound temperature, which do not directly capture critical immune dynamics such as immune cell function, inflammatory pathways, and redox balance. This limitation restricts the AI’s ability to guide precise treatment decisions, such as when to initiate anti-inflammatory interventions or promote angiogenesis and ECM remodeling. From a translational perspective, AI strategies that focus on real-time wound monitoring, risk stratification, and clinician-oriented decision support currently exhibit the greatest developmental potential, as they can be readily embedded into existing clinical workflows and evaluated using conventional clinical endpoints. To overcome this challenge, future AI models must integrate real-time data reflecting immune microenvironment evolution, allowing for more accurate and timely treatment planning, while maintaining clinician oversight to ensure safety, interpretability, and regulatory feasibility.

The future success of diabetic CWs treatment depends on the deep integration of smart nanocarriers with AI systems. First, nanocarrier design should prioritize multi-signal responsive systems, where modular and hierarchical responses enable precise matching of therapeutic interventions with wound healing stages. For example, AI could dynamically adjust the drug release strategy of nanocarriers based on real-time monitoring of immune responses, ensuring timely intervention for each healing phase. Second, AI’s role must evolve from static imaging analysis to more comprehensive immune state quantification. By continuously monitoring immune dynamics, oxidative stress levels, and immune cell polarization, AI can provide crucial data to optimize drug release timings and dosages. This integration of immune modulation and real-time data-driven decisions will create a more controlled and predictable approach for diabetic CWs treatment, making AI and smart nanocarriers a promising combination for personalized therapies. From a near-term clinical implementation perspective, the principal constraints are more closely associated with prolonged regulatory approval processes, clinical trial requirements, and long-term safety validation, rather than with inherent risks of AI technologies per se. Based on the current body of preclinical evidence and the limited but gradually accumulating early-stage clinical data, AI-enabled wound monitoring and treatment optimization tools are more likely to enter clinical practice in the short term as adjuncts to existing care paradigms, whereas fully autonomous closed-loop therapeutic systems will require substantially longer periods of validation and regulatory standardization.

## Figures and Tables

**Table 1:** Core Pathological Features and Immune Consequences in Diabetic Chronic Wounds with Therapeutic Targets.

Pathological Feature	Key Molecular Mechanisms	Immune Consequences	Therapeutic Intervention Opportunities	Ref
Hyperglycemia & AGEs accumulation	AGEs–RAGE–NF-κB activation; ECM cross-linking	Chronic inflammation; M1 macrophage polarization; impaired angiogenesis	Localized delivery strategies targeting the AGEs–RAGE axis to disrupt chronic inflammation and oxidative stress	[20]
Excessive oxidative stress	ROS overproduction; antioxidant system suppression (SOD, GSH-Px)	Inhibition of M2 polarization; NETs overproduction; endothelial injury	ROS-scavenging nanozymes; redox-responsive hydrogels	[21,22]
Microvascular dysfunction	Sustained activation of the NLRP3 inflammasome; PFKFB3-dependent glycolysis inhibition	Impaired neovascularization; hypoxia-driven inflammation	Pro-angiogenic and metabolic-regulating nanocarriers to improve endothelial function and immune response	[23,24]
Peripheral neuropathy	Reduced CGRP, substance P, NGF signaling	Disrupted neuro-immune-vascular crosstalk	Neurotrophic factor-loaded smart patches and conductive hydrogels	[25]
Abnormal ECM remodeling	MMP overactivation; collagen I/III imbalance; ECM stiffening	Persistent ECM degradation and delayed resolution of inflammation	MMP-responsive biomaterials to modulate ECM degradation and deposition for tissue regeneration	[26,27]

**Table 2:** Representative immune-regulating smart composite nanocarriers for diabetic CWs in response to pathological microenvironmental cues.

Carrier Type	Nanocarrier	Pathological Trigger	Advantages	Ref
ROS-responsive	Manganese dioxide nanoflower hybridized gold nanoparticle composites (MnO_2_-Au nanoflowers)	Excess ROS	Efficient H_2_O_2_ cleavage; enhanced ROS scavenging; improved wound healing with collagen deposition.	[82]
ROS-responsive	Quaternized chitosan/salvianolic acid B (QF/SAB) hydrogel	Glucose, excess ROS	Dual-responsive to ROS and glucose; promotes M2 macrophage polarization; accelerates wound healing by reducing inflammation and enhancing angiogenesis.	[102]
ROS-responsive	Clindamycin@Nanoceria (CLIN@CNP)	Inflammation-associated ROS	Scavenges ROS; antibacterial effects against Gram-positive and Gram-negative bacteria; enhances wound healing.	[103]
pH-responsive	N, O-carboxymethyl chitosan/oxidized hyaluronic acid (N, O-CMCS/OHA) nanogels	Excess ROS, bacterial infection, high glucose levels	pH/HAase dual-responsive release for targeted delivery, antibacterial activity; accelerates wound healing.	[86]
pH-responsive	Carboxymethyl chitosan/2-formylphenylboronic acid/tannic acid-Fe (CMCS/2-FPBA/TA-Fe) hydrogel	High glucose, bacterial infection, acidic pH	pH-responsive; antibacterial; promotes wound healing; reduces inflammation; stimulates angiogenesis; promotes M1 to M2 macrophage polarization.	[104]
pH-responsive	Alginate/copper (Alg/CuP hydrogel	Acidic (infection phase)/alkaline (repair phase)	POD activity in acidic conditions; CAT activity in alkaline conditions, enhances antibacterial and angiogenic properties.	[105]
Enzyme-responsive	Galactose-modified PLGA nanospheres (F@GP)	Elevated H_2_O_2_ in senescent cells	Targets senescent cells with Fe_3_O_4_; induces ferroptosis via Fenton reaction; promotes wound healing by enhancing re-epithelialization and cell proliferation.	[91]
Enzyme-responsive	Prussian blue nanoparticles (PBNPs) encapsulated in gelatin nanospheres (PGs)	Elevated MMP-2/MMP-9	Smart release of PBNPs to clear ROS, regulate macrophage polarization (M1 to M2), and enhance wound healing.	[106]
Macrophage polarization-responsive	4′-Hydroxychalcone (4HC)-loaded nanofiber hydrogel	TLR/IL-17/TNF signaling pathway	Inhibits TLR/IL-17/TNF pathway; promotes M2 macrophage polarization; reduces inflammation; enhances diabetic wound healing.	[92]
Macrophage polarization-responsive	Ginseng-derived nanoparticles (GDNPs)	Excess ROS, high glucose, inflammation	Promotes M2 macrophage polarization; reduces inflammation; enhances angiogenesis; activates PI3K/AKT/HIF-1α; inhibits TLR4/MyD88/MAPK pathways for wound healing.	[107]
Growth factor-immune modulator nanocarriers	Dermal matrix hydrogel (HDM) loaded with VEGF and IL-10 microspheres	Chronic inflammation, impaired angiogenesis	Controlled release of VEGF and IL-10; promotes angiogenesis; reduces inflammation; enhances collagen deposition; accelerates wound healing.	[95]
Growth factor-immune modulator nanocarriers	Growth factor coated BSA nanoparticle embedded nanofibers (GF-NP-NFs)	Growth factor depletion	Sustained VEGF/bFGF delivery to enhance regenerative signaling.	[108]
Stem cell-loaded nanocarriers	Wharton’s Jelly mesenchymal stem cells (WJMSCs) encapsulated in hydroactive gel	Chronic inflammation	Accelerates diabetic wound healing; promotes macrophage polarization; enhances cell proliferation, angiogenesis, dermis regeneration, and stem cell retention.	[99]
Stem Cell-loaded nanocarriers	Hydrogel loaded with oxygen-releasing microspheres and CDCs	Hypoxic inflammatory microenvironment	Alleviates hypoxia to improve stem cell survival and therapeutic durability	[109]

## Data Availability

No new data were created or analyzed in this study.
